# Can the artificial exogenous addition really cause an increasing carbon emission driven by microbial community in grassland ecosystems?

**DOI:** 10.3389/fmicb.2024.1421325

**Published:** 2024-07-04

**Authors:** Guanhong Liu, Ze Gu, Bingyi Li

**Affiliations:** ^1^Yinshanbeilu Grassland Eco-Hydrology National Observation and Research Station, China Institute of Water Resources and Hydropower Research, Beijing, China; ^2^School of Ecology and Nature Conservation, Beijing Forestry University, Beijing, China; ^3^Hebei Normal University, Shijiazhuang, Hebei, China

**Keywords:** rhizosphere priming effect, exogenous additions, soil carbon emissions, grassland ecosystems, microbial community dynamics

As anthropogenic impacts intensify under the background of global change, the response of terrestrial ecosystems to human-induced stressors and climatic shifts has become an increasingly focal area of research (Tanentzap and Kolmakova, 2023). Exogenous amendments to soil, particularly the incorporation of water and organic carbon sources, represent a significant facet of global change dynamics (Zia et al., 2021; Liu et al., 2023). Such global transformations can enhance the influx of moisture and organic substrates into certain soils, instigating alterations in the carbon cycling rates of microbial assemblages, known as the “priming effect” (Kuzyakov et al., 2019). The priming effect refers to the change in the rate of microbial decomposition of soil organic matter (SOM) due to the addition of external substrates, such as water and organic carbon. Under different environmental conditions, this effect can manifest in various ways. For example, in nutrient-poor or arid soils, the introduction of water and organic carbon can significantly stimulate microbial activity, leading to an accelerated decomposition of SOM. Conversely, in nutrient-rich or moist soils, the priming effect may be less pronounced, as microbial communities are already active. Predominantly positive, this priming effect, induced by the external addition of water and organic carbon, is believed by many scholars to foster a positive feedback mechanism in the context of global change (Pausch and Kuzyakov, 2018). This feedback loop can result in increased CO_2_ emissions from soils, thereby contributing to further climate change. Over the long term, the priming effect can alter the balance of carbon cycling processes, influencing soil carbon storage and atmospheric carbon levels. Grassland ecosystems, characterized by more arid and nutrient-poor soils compared to forest ecosystems, are purportedly more prone to extensive positive priming effects. Consequently, some research posits that exogenous amendments in grasslands could greatly amplify soil carbon loss, thereby hastening the progression of climate change (Luo et al., 2016). However, the veracity of this assertion warrants further examination. Understanding the ecological significance of the priming effect, particularly its role in the broader context of global changes, is crucial for predicting and mitigating the impacts of anthropogenic activities on terrestrial carbon dynamics.

Through existing articles, we have identified considerable uncertainty regarding whether exogenous additions to grassland ecosystems result in large-scale soil carbon release. Initially, the concept of the priming effect was based on laboratory experiment results; however, the substantial differences between laboratory and field conditions make it imprudent to extrapolate laboratory findings directly to field scenarios (Song et al., 2017b). Due to these substantial differences, some researchers have started using deep learning techniques to better understand and predict field conditions based on laboratory data. These advanced techniques, including convolutional neural networks and other machine learning methods, can help bridge the gap between controlled experiments and real-world applications by analyzing large datasets and identifying complex patterns (Tetila et al., 2020). Additionally, the priming effect is often a short-term phenomenon, and the immediate responses of microbes do not necessarily reflect their behavior over longer periods, thus casting doubt on the presence of a consistently positive priming effect under field conditions (Bernard et al., 2022). For instance, recent research has shown that the effects of drought intensity on priming effects and their temperature sensitivity vary significantly, indicating that microbial responses to environmental changes are complex and context-dependent (Zhang R. et al., 2024). Most importantly, if the carbon released by microbes—including both net rhizodeposition and the decomposition of dead roots—is humified into the soil organic carbon content, exceeding the additional carbon release caused by the positive priming effect, then the total soil carbon stock should increase (Sun and Zhu, 2023). Most studies on the priming effect focus solely on carbon release without considering the overall carbon budget (Bernard et al., 2022). Therefore, we have grounds to believe that exogenous additions to grassland ecosystems are unlikely to cause large-scale soil carbon release.

## Evidence from soil respiration components

**Figure 1:**
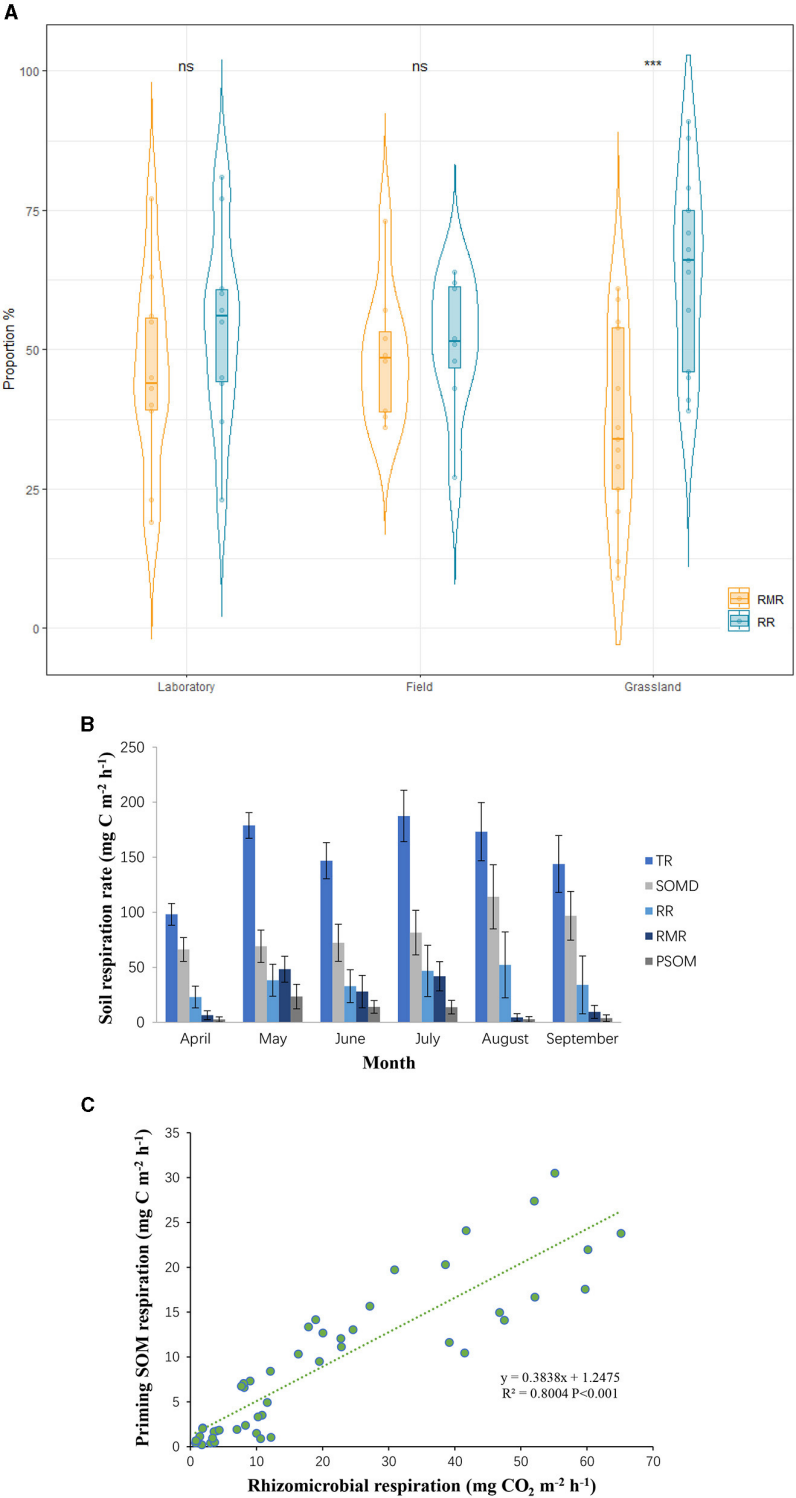
**(A)** Contribution rate of root and rhizo-microbial respiration to rhizosphere respiration. **(B)** Seasonal variation of the rates of total soil respiration (TR), decomposition of soil organic matter (SOMD), root respiration (RR), rhizo-microbial respiration (RMR), and the priming decomposition of soil organic matter (PSOM). **(C)** Relationship between RMR and PSOM. ns means not significant. ***means extremely significant (*p* < 0.001).

The rhizosphere priming effect, defined as the stimulation of rhizosphere microorganisms by carbon inputs from root deposition, is the predominant priming effect in grassland ecosystems (Li et al., 2022). Rhizosphere microbial respiration (RMR), which is the release of CO_2_ by rhizosphere microorganisms as they decompose root-deposited carbon, shows a positive correlation with the rhizosphere priming effect (Zhu et al., 2014). This effect occurs because rhizodeposited carbon, such as root exudates and decaying root material, provides an energy source for soil microorganisms. The influx of fresh organic matter stimulates microbial activity, enhancing their decomposition processes and accelerating soil organic matter turnover. This increased microbial activity and decomposition can lead to higher CO2 emissions, contributing to the overall soil carbon dynamics (Lei et al., 2023). Both RMR and autotrophic root respiration (RR), which utilize organic matter produced by plant photosynthesis, are considered components of autotrophic respiration, and thus are not included in the net increase in carbon emissions (Song et al., 2017b). Overall, in grassland ecosystems, RMR and RR constitute 36% and 64% of autotrophic respiration, respectively, with RMR significantly lower than RR (Figure 1A). However, comparisons between laboratory and field data reveal that laboratory results significantly overestimate RMR (Figure 1A), indicating an overestimation of the scale of the priming effect in laboratory experiments.

Furthermore, heterotrophic respiration driven by the priming effect, which truly contributes to the net increase in carbon release, is referred to as primed soil organic matter (PSOM) (Werth and Kuzyakov, 2010). If exogenous additions to the rhizosphere were to cause large-scale carbon release, then PSOM would constitute a significant proportion of total respiration. Yet, the reality is that RMR is significantly positively correlated with PSOM, and RMR is consistently higher than PSOM, regardless of conditions (Figure 1B). Results from a single growing season also demonstrate that PSOM accounts for < 5% of total carbon emissions (Figure 1C). This indicates that exogenous additions merely cause a minor decomposition of soil organic matter by microorganisms utilizing the added organic substrates, and this slight increase in carbon release does not lead to substantial positive feedback to the atmosphere (Song et al., 2017a). These findings are significant for managing grassland ecosystems, as they suggest that exogenous additions are unlikely to cause substantial carbon loss. This has important implications for studying and mitigating the effects of global changes on soil carbon dynamics.

## Evidence from field experiments

The majority of experiments examining the effects of exogenous additions on soil microbial activity are conducted in laboratories, where results often substantially differ from field conditions, making *in situ* field experiment findings more credible. Some field control experiments have attempted to demonstrate that exogenous additions increase carbon emissions due to enhanced microbial decomposition activity by adding glucose (Zheng L. et al., 2024). However, the problem is that glucose is an exceedingly rare addition in natural settings. For instance, studies in the Inner Mongolia grasslands show that the main exogenous additions to plant roots are lipids and polysaccharides, with monosaccharides being almost nonexistent (Yu et al., 2024). In fact, adding non-monosaccharide organic matter does not enhance microbial decomposition; rather, it increases the production of recalcitrant organic matter by microbes, enhancing the efficiency of the 'microbial carbon pump' and thereby promoting soil carbon sequestration (Liang and Zhu, 2021; Song et al., 2023). For example, new findings indicate that changes in root exudate inputs in grassland ecosystems only affect the composition of rhizosphere microorganisms, particularly the composition of fungi, and do not affect the carbon pool of non-rhizosphere soil (Yu et al., 2024). The increase in fungi caused by the increase in root exudates leads to an increase in wall/membrane/envelope biogenesis, which increases the content of microbial residues, thereby promoting soil carbon sequestration (Song et al., 2023). A meta-analysis based on 1,272 sets of experimental data from around the globe also shows that the soil priming effect changes from a positive to a negative effect as the addition of external sources increases (Xu et al., 2023).

Furthermore, the duration of exogenous additions is a critical factor influencing the priming effect. If additions are only made briefly, the priming effect appears temporarily and quickly subsides (Zhou et al., 2022). However, continuous long-term exogenous additions do not generally result in sustained high levels of carbon release in field studies. Long-term carbon addition and water augmentation experiments indicate that prolonged exogenous additions do not significantly impact the soil microbial community (Kuzyakov et al., 2019; Yang et al., 2024). This is because ecosystems possess inherent mechanisms to maintain homeostasis. One such mechanism is the resilience of microbial communities, which can adapt to changing conditions by altering their composition and function to utilize available resources efficiently (Shade, 2023). Additionally, soil buffering capacity helps maintain pH and nutrient levels, preventing drastic shifts in soil chemistry that could disrupt microbial activity. Plant-microbe interactions also contribute to homeostasis, as plants can modulate root exudate production to influence microbial communities and stabilize the soil environment (Chaudhry et al., 2021). Besides, feedback loops between soil microorganisms and plant roots help regulate nutrient cycling and carbon flow, ensuring that any initial perturbations caused by exogenous additions are mitigated over time (Yan et al., 2022). Thus, long-term additions merely transition the ecosystem into a new steady state without causing significant disruptions (Yu et al., 2021). For example, new research indicates that different plant species in grassland communities can alleviate soil nutrient limitations through reabsorption and maintain the stability of their communities (Zheng Y. et al., 2024). Another study shows that the soil microbial community in grasslands undergoes significant changes 1 year after initial disturbance, but returns to its original state by the 2^nd^ year (Yu et al., 2021). Additionally, research has demonstrated that mycorrhizal fungi (especially ectomycorrhizal fungi) can significantly enhance ecosystem stability, thereby maintaining the steady state of soil ecosystems (Guo et al., 2024). These homeostatic mechanisms enable ecosystems to absorb and adapt to changes, ensuring that long-term exogenous additions result in a stable new equilibrium rather than significant disruptions. This understanding is crucial for evaluating the long-term impacts of exogenous additions on soil carbon dynamics and for developing effective management strategies for grassland ecosystems.

## Conclusion

Substantial uncertainty persists regarding whether exogenous additions to grassland ecosystems result in significant soil carbon emissions. The profound environmental disparities between laboratory settings and field conditions, along with the nature and duration of exogenous additions, can lead to misinterpretations of research outcomes. Both the components of soil respiration and evidence from *in situ* field experiments do not support the hypothesis that the priming effect causes significant positive feedback from the grassland soil microbial community to the atmosphere. Therefore, it is our contention that exogenous additions to grassland ecosystems are unlikely to lead to large-scale soil carbon emissions.

However, several questions remain unanswered and warrant further investigation. How do different types of organic substrates influence the priming effect under varying environmental conditions? What are the long-term impacts of repeated exogenous additions on soil carbon sequestration and microbial community dynamics? Future research should also explore the role of plant-microbe interactions in modulating the priming effect and how these interactions may shift under changing climate conditions. Addressing these questions will be crucial for developing a more comprehensive understanding of the mechanisms driving soil carbon dynamics in grassland ecosystems and for informing sustainable management practices.

## Author contributions

GL: Writing – review & editing, Writing – original draft, Software, Methodology, Investigation, Funding acquisition, Data curation. ZG: Writing – review & editing, Methodology, Investigation, Data curation. BL: Writing – review & editing, Validation, Resources, Project administration, Data curation.
